# Supplementary material for: Natural hybridization in heliconiine butterflies: the species boundary as a continuum
Source: BMC Evol Biol. 2007 Feb 23;7:28. doi: 10.1186/1471-2148-7-28 (PMC1821009; doi:10.1186/1471-2148-7-28)
Supplement: Additional File 1 — Hybrids between species of Heliconius and Eueides butterflies: a database. HTML file linking to database of all known wild-caught interspecific hybrid specimens in the Heliconiina, consisting of introductory text, a list of specimens, together with collection data and photographs of the specimens, and links to information about some artificial hybrids and mutants in the group. This is an edited copy of our online database of Heliconius hybrids [102]. To view database, download zip file and extract to a separate folder, then open index.html within that folder. [file 1471-2148-7-28-S1.zip › mutants/mutants.html]

Mutants and other bizarre Heliconiina


# Mutant and aberrant *Heliconius* and *Eueides*


## See also: ***Heliconius* hybrids**


---

Some bizarre
wild-caught or laboratory specimens of *Heliconius* and heliconiine butterflies
thought to be mutants or phenotypic aberrations (including homeotics) rather
than hybrids. 

|  |  |
| --- | --- |
|  | An odd-looking *Eueides* ("New Granada, Hewitson coll., *Eueides edias*" -- NHM, London) |
|  | *Neruda aoede* - forewing-bandless mutant (Yurimaguas, Peru, Dec 2005, Mathieu Joron) |
|  | *Heliconius melpomene*, crossover within *D^R^* gene (Tarapoto Peru, 1986, J. Mallet [1989] Proc. Roy. Soc. 236:163-185, Plate 2 Fig. 4) |
|  | *Heliconius erato*, crossover within *D^R^* gene (Tarapoto Peru, 1986, J. Mallet [1989] Proc. Roy. Soc. 236:163-185, Plate 2 Fig. 4) |
|  | *Heliconius cydno* x *H. melpomene* interspecific hybrid, gynandromorph, artificially reared (Austin, Tx., 2001, L.E. Gilbert) |
|  | *Heliconius charithonia bassleri* aberration (Colombia, Luis Miguel Constantino) |
|  | *Heliconius erato* homeotic (Sirena, Corcovado, Costa Rica 1981, J. Mallet).  The right hindwing upperside is normal, but the overall shape of the hindwing, as well as the venation, is distorted by the partial homeosis on the underside. I was involved in a mark-release-recapture study, and had marked the left side in the field with the number 818, turned the butterfly over, and realized that the right side was homeotic.  In retrospect, I should have bred from this beast to try to get a homeotic mutant, though it was almost certainly purely somatic. |
|  | *Heliconius erato*, crossover within *D^Ry^* gene (Tarapoto Peru, 1986, J. Mallet [1989] Proc. Roy. Soc. 236:163-185, Plate 2 Fig. 4) |

---

Source: J.
Mallet    
Back to: Heliconiine hybrids   
**Last updated:** 7 August 2006
